# The Impact of Surface Preparation for Self-Compacting, High-Performance, Fiber-Reinforced Concrete Confined with CFRP Using a Cement Matrix

**DOI:** 10.3390/ma13122830

**Published:** 2020-06-24

**Authors:** Krzysztof Adam Ostrowski, Roman Kinasz, Piotr Dybeł

**Affiliations:** 1 Institute of Building Materials and Structures, Cracow University of Technology, 24 Warszawska Str., 31-155 Cracow, Poland; 2 Faculty of Mining and Geoengineering, AGH University of Science and Technology, 30 Adama Mickiewicza Str., 30-059 Cracow, Poland; rkinash@agh.edu.pl (R.K.); dybel@agh.edu.pl (P.D.)

**Keywords:** concrete surface, CFRP, cement matrix, high-performance self-compacting fiber reinforced concrete, stress-strain characteristic, reinforcement

## Abstract

With the development of concrete technology, the tendency to combine different materials with each other to achieve a greater efficiency and durability of structures can be observed. In the modern construction industry, various materials and techniques are increasingly being combined in order to achieve e.g., an increased resistance to dynamic impacts of a structure, or an increased scope of work of a selected constructional element, which translates into a significant increase in the energy of destruction. Thus, hybrid elements, known as composite ones, are created, which consist of concrete and reinforcements. This study examined the influence of the preparation of the concrete surface on the behavior of high-performance, self-compacting, fiber-reinforced concrete (HPSCFRC), reinforced with carbon fibers (CF) using a cement matrix. In the general lamination processes, this is preformed using epoxy resin. However, epoxy resin is sensitive to relatively low temperatures, and therefore the authors attempted to use a cement matrix in the lamination process. When connecting hardened concrete with a fresh concrete matrix or mixture, the type of the concrete surface is significant. In this research, three types of concrete surfaces e.g., unprepared, sanded and grinded were considered. All of the surfaces were examined using a 3D laser scanner, to determine the Abbott-Firestone profile material share curve. In this research, cylindrical concrete specimens were reinforced with one, two and three layers of laminates. They were then subjected to a uniaxial compressive test. The results of tests showed that the use of cement matrix in the lamination process, due to its low efficiency, should not be applied when reinforcing concrete elements with a high compressive strength. Moreover, the grinded surface of concrete showed the best cooperation with CF reinforcement.

## 1. Introduction

Composites (combined materials) are materials that are consciously constructed of two or more materials and which have properties other than individual constituent materials. Fiber reinforced polymer (FRP) composite can be defined as a polymer which is reinforced with fiber. The use of FRP in civil engineering is becoming more and more common in relation to strengthening existing and newly designed structures. This is due to the many advantages of materials from this group of polymers. The most important advantages of FRP materials are its: high Young modulus, strength to weight ratio, resistance to aggressive environments, good fatigue properties, low lifecycle costs, electromagnetic transparency and low thermal conductivity [1,2,3,4,5,6,7]. 

In civil engineering, intensive growth in the design of structures manufactured by self-compacting concrete (SCC) has been observed [8,9]. The use of classic unmodified concrete in some types of building structures is not sufficient today. Requirements for structures in terms of bearing capacity and serviceability are increasing, and their fulfillment using ordinary concrete becomes uneconomical and even impossible in some cases. This forced the search for new ways to improve the properties of concrete. Thanks to the development of material engineering, a “new class of concrete” has emerged called fiber-reinforced concrete [10,11]. A high compressive strength, characteristic rheological properties of the fresh concrete mixture and quasi-plastic stress-strain curve are specific for high-performance self-compacting fiber-reinforced concrete (HPSCFRC). The usage of steel fiber into the structure of concrete is essential to obtain high ductility and strength of this specific kind of construction material [12,13,14,15,16,17]. 

Carbon fibers (CF) are the most often used to strengthen engineering structures, and are characterized by good thermal and chemical resistance. Working temperature is one of the most important criteria for choosing a given type of fiber to reinforce a composite material. For example, the properties of carbon fibers do not change in a non-oxidizing atmosphere to 2000 °C, unlike glass or aramid fibers [18,19]. Carbon fibre reinforced polymer (CFRP) materials are commonly used to reinforced variety type of structures in civil engineering. However, this technique is a relatively expensive but effective solution [20]. In case of concrete studies, CFRP is often considered as an effective and alternative technique to reinforce many types of concrete or masonry structures incorporating old structures and monuments [21,22,23]. In general, the current literature contains a lot of research that relates to concrete reinforcement with CFRP in various types of samples, such as classic columns [24], elements with CFRP material in cavities of concrete surfaces [25] and concrete columns under eccentric load [26]. The development of CFRP techniques can also be observed in case of beam structures. Zaki et al. [27] proposed CFRP fiber anchors as a reinforcement of T-shaped beams. Zhang et al. [28] proposed the use of stirrups from CFRP to reinforce concrete beams made of reinforced aggregate concrete from recycled aggregate. Researchers tested reinforced concrete using various types of FRP. Kissman and Sundar examined reinforced concrete column with one and two layers of glass fiber reinforced polymer (GFRP). Obtained results has been shown that compressive strength and axial strain of reinforced specimens were greater in compare to plain concrete [29]. Sadeghian and Fillmore reinforced concrete specimens with basalt fiber reinforced polymer (BFRP). When reinforcing concrete with two, four and six layers of BFRP, increased strength factor of 1.41, 1.92 and 2.36 was obtained, respectively [30]. Campione et al. has been shown that using BFRP stabilization of post-peak behavior of reinforced concrete could be reached [31]. Aramid fiber reinforced polymer (AFRP), as the confinement of concrete, was studied by Toutanji and Deng [32]. According the authors, dry or wet environment does not have a significant impact on the strength of AFRP reinforced concrete specimens. In 2017, Eid and Paultre proposed the stress-strain model to predict the behavior of concrete reinforced with FRP composites [33].

CFRP still remain a new tendency in the design process. The role of the matrix in the laminate can be played by epoxy resin, and sometimes by the cement matrix [34]. Due to the plasticization of the resin at relatively low temperatures, alternative matrices are being sought for reinforcing concrete structures. In some cases, this role can be played by the cement matrix, which is definitely more resistant to the effects of elevated temperatures. The usage of combining composite materials and inorganic matrices could affect the strengthening of reinforced concrete [35]. The cement matrix is most often made using CEM I 52.5R cement, silica fume, water and a large amount of a new generation superplasticizer (up to 10% of the cement mass). The effective time for using the cement matrix in FRP laminates is usually no more than 30 min. The major advantages of using cement matrix in lamination process of fiber reinforced materials (FRP) are: high compressive strength (to 100 MPa), no plastic behavior at high temperatures, low cost, high stiffness and higher resistance to high temperatures with epoxy resins. On the other hand, low tensile strength (to 10 MPa), chemical resistance and bending strength are the major drawbacks.

It has been proven many times that CFRP can protect the structure of concrete [36] and composite structures [37]. However, the use of cement matrix for reinforcing concrete elements in FRP technique depends on many aspects such as concrete strength and FRP type [38]. According to Al-Abdwais, the debonding of FRP reinforcement from the concrete surface could be a crucial failure in the external bond, which could lead to a reduction of the bond strength [39]. It has been shown that flexible and stiff adhesives can be effectively used in strengthening concrete structures with CFRP, where the near-surface mounted (NSM) technique is considered [40,41]. The matrix material in case of FRP composites has crucial functions: to protect the FRP and stresses transfer between FRP and the matrix [42]. Existing studies have demonstrated that bond behavior between CFRP and concrete depends on the type of concrete and the roughness of the concrete surface [43,44]. 

## 2. Research Significance

In most of the analyzed literature, researchers do not provide detailed information on surface preparation, and often provide basic information that the concrete surface has been cleaned and dusted before the lamination process. Only a few works provide information concerning the method of preparing a concrete surface. In most cases the surface was not prepared using any technological treatment, or was treated using grinding. The lack of morphological characteristics of the concrete surface in many scientific papers inspired the main author of this work to analyze the impact of surface preparation on the efficiency of reinforcing concrete elements with carbon fibers. Due to the plastic behavior of epoxy resins in low temperatures, the authors tried to answer the question of whether a cement matrix could be reasonably used in the lamination process of high-performance concrete. The paper analyzes three types of the most common concrete surfaces: unprepared, sanded and grinded. In summary, the main purpose of the work is to indicate the optimal concrete surface intended for reinforcing when using FRP materials.

## 3. Materials and Methods

### 3.1. Preparation of Concrete Mixtures

**Figure 1:**
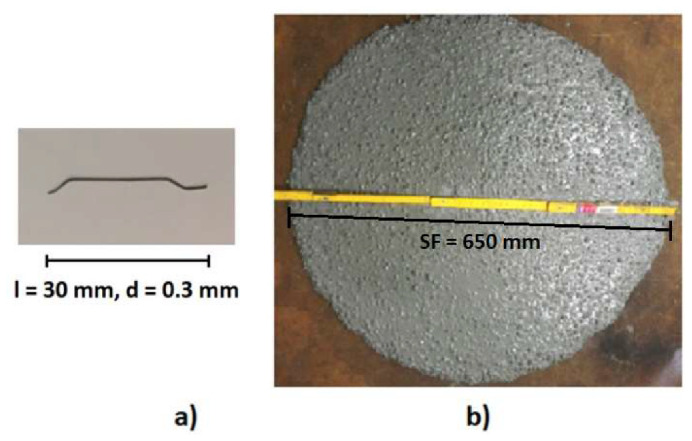
Steel reinforcement (**a**) and the slump flow test for the concrete mixture (**b**).

The HPSCFR concrete mixture was applied in this research. The materials which were used to create the mixture were as follows: Portland Cement type I 52.5 R with a rapid increase in strength (500 kg/m^3^), river sand as fine aggregate (650 kg/m^3^), diabase as coarse aggregate (1000 kg/m^3^), superplasticizer Sikament FM6 (17.5 kg/m^3^), Sika Fume addition as microfiller (60 kg/m^3^) and water from waterworks (160 kg/m^3^). The water to cement (w/c) ratio was equal to 0.32. The steel micro-reinforcement (78 kg/m^3^) had the form of steel oar-shape fibers with a length (l) of 30 mm and an outer diameter (d) of 0.3 mm (Figure 1a). The tensile strength of the steel fibers, according to the manufacturer (Urban-Metal Sp. z o.o., Rachowice,, Poland), was equal to 1100 MPa. The slump flow (SF) test was used to determine the fresh properties of the HPSCFR concrete mixture. The slump flow was equal to 650 mm (Figure 1b) and the plastic viscosity was 12.5 s, which were defined according to European Standards [45]. Separation of the concrete mixtures’ components was not observed.

### 3.2. Specimens

A total of 40 specimens were analyzed in the presented studies. All cylinder specimens were 200 mm in height and had an outer diameter of 100 mm. All columns were divided into three groups: 12 samples were sanded (S) and grinded (G) in each group, and the remaining 12 samples had an unprepared surface (U). The samples were then reinforced with 1, 2 and 3 layers of carbon fibers—4 samples for each series. The samples were marked as follows: U/S/G-1:3-1:4. For example, S-2-4 means a fourth concrete sample with a sanded surface, reinforced with two layers of carbon fibers using cement matrix. Samples S-1, S-2, S-3 and S-4 are the reference samples, and not reinforced in any way.

### 3.3. Reinforcing of Concrete with Carbon Fibres

A carbon fiber (CF) overlap of 50 mm was provided for each sample. The properties of the CF according to manufacturer are as follow: density (304 g/m^2^), effective thickness (0.167 mm), ultimate elongation at the break (1.7%), tensile strength (4900 MPa) and Young’s modulus (230 GPa). In the case where the samples were reinforced with more than one layer, a pre-cut CF mat was prepared, to ensure the appropriate length of the reinforcement. For samples reinforced with one layer, 364 mm of CF reinforcement was used. For two and three layers, 678 mm and 992 mm were used, respectively. The height of the CF was 200 mm. The specimens were laminated with high performance cement matrix during the dry lay-up process. The content of the cement matrix was as follows (by mass): cement type I 52.5 R: Sika Fume HR: Water: Plasticizier Sikament FM 6, which was equal to 100:30:40:10. The compressive strength of the cement matrix was 64 MPa. Firstly, the whole concrete sample was embedded in the cement matrix, and then a CF mat was applied to the concrete surface, which was covered with a cement matrix using a brush, while trying to soak and infiltrate the CF. The thickness of the cement matrix was about 4 mm. The lamination process was carried out at a temperature of 24 °C, and a humidity of 65% ± 5%. The samples reinforced with the cement matrix were tested 28 days after lamination.

### 3.4. Instrumentation and Testing Procedure

**Figure 2:**
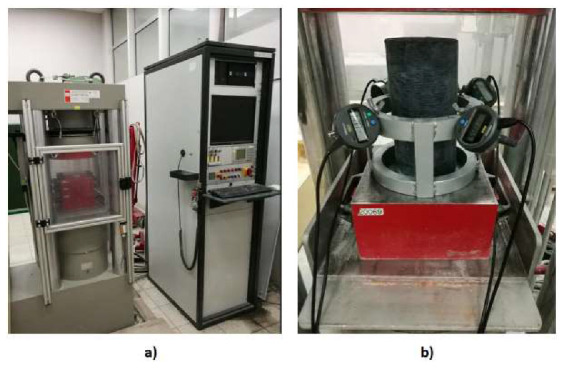
Compressive testing machine Walter + Bai AG (**a**) and localization of transverse strain gauges (**b**).

The samples were subjected to a uniaxial compression test using a 3000-kN capacity testing machine (Figure 2a, Walter + Bai AG, Löhningen, Switzerland). All the tests were carried out in a local Laboratory of Concrete Structures, in AGH, Cracow, Poland, in a humidity of 50 ± 5% and an air temperature of 20 ± 1 °C. The constant axial force rate, equal to 5 kN* s^−1^, was provided during the compression test. The instrumentation and testing procedure were in accordance with norm [46]. The axial displacement, force value and test time were read from a text file. The text file is a record of the test parameters for each sample. This means that the axial displacement values are the displacement values of the testing machine piston during the testing of the elements. The transverse displacements were measured using extensometers located at half the height of the samples using four electronic sensors spaced 90° apart, with an accuracy of 1 × 10^−3^ mm. The test setup, illustrating the transverse strain gauges applications, is presented in Figure 2b. 

## 4. Morphological Characterization of a Concrete Surface

### 4.1. D Laser Scanner

The characteristics of the concrete surfaces were determined by scanning the surface. Concrete surface scanning was performed using an innovative 3D laser scanner (Wrocław University of Science and Technology, Wrocław, Poland) based on the method of laser triangulation, which involved measuring the deformation of the line produced by the laser beam. The distances between individual points located on the tested surface were measured with a stepper motor-driven camera, thanks to which, measurements can be made with an accuracy of 15 µm in profiles spaced 10 mm apart. The laser has a compact structure, and therefore tests can be carried out in both laboratory and field conditions. The device assigns three coordinates to each measuring point, describing its location on the test surface. The measurement data file is saved in asc format. The result of the scan is a virtual 3D image of the morphology of the surface being examined. The image was analyzed in order to obtain the values of the parameters that describe the surface morphology [47].

### 4.2. Description of the Concrete Surface

The profile material share curve is the curve that illustrates the profile material share at a certain level c (most often expressed as a percentage) as a function of distance c from the line passing through the highest point of the profile, and which is parallel to the average line. This curve is also called the Abbott-Firestone curve [48,49,50,51]. Thanks to the shape of the curve of the material share of the profile, it is possible to analyze the surface resistance of a given material to tribological wear. The shape of the material share curve is determined using parameters determined on its basis [24]. The following areas can be distinguished in the material share curve area:

- Profile elevation zone;
- Profile core zone;
- Profile recess zone.

**Figure 3:**
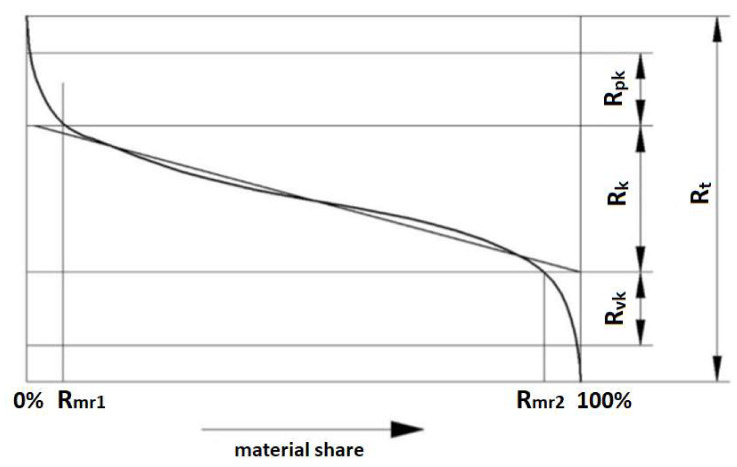
Rough material profile curve and its parameters.

The following parameters of the material share curve of the surface roughness profile are distinguished (Figure 3):

- Average height of high mid elevations above the core of the roughness profile—reduced height of R_pk_;
- The depth of the middle part of the profile, determined by a simple linearizing material share curve—depth of the roughness core R_k_;
- Average depth of deep pits under the roughness core—reduced depth of R_vk_ pits.

The material fraction R_mr1_ corresponds to the upper limit of the core of the profile, while the material fraction R_mr2_ corresponds to the lower limit of the core. The R_t_ is the distance between the highest and the lowest point on the surface roughness profile.

### 4.3. Preparation of the Specimens

**Figure 4:**
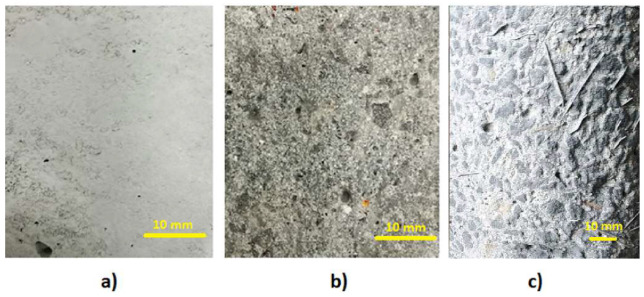
Unprepared surface (**a**), grinded surface (**b**) and sanded surface (**c**).

**Figure 5:**
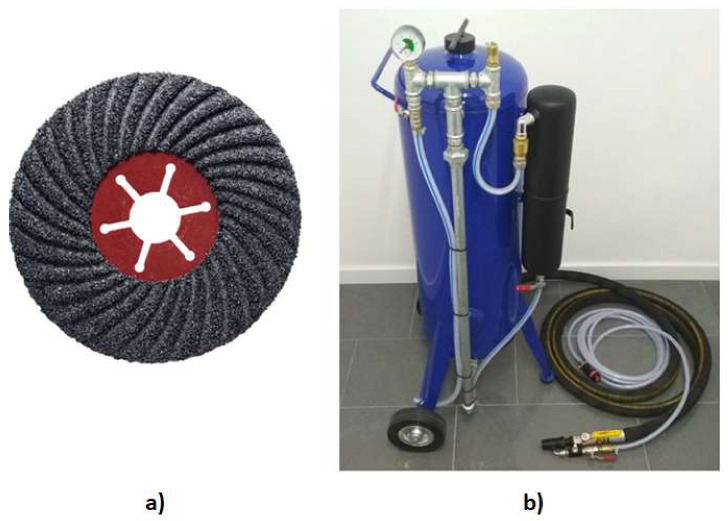
Ceramic grinding disc (**a**) and siphon blasting machine with a water nozzle (**b**).

In order to characterize the surface of the concrete, an additional three cubic samples of HPSCFRC with dimensions equal to 10 cm × 10 cm × 10 cm were made. The first sample had an unprepared surface (sample U1—Figure 4a). The second cube was grinded (sample G1—Figure 4b) and the third one was sanded (sample S1—Figure 4c) in the same manner as the cylinder specimens. The grinded surface was prepared using a ceramic grinding disc (Figure 5a), while the sanded surface was made using a siphon sander with a water nozzle (Figure 5b). 

### 4.4. Results

**Figure 6:**
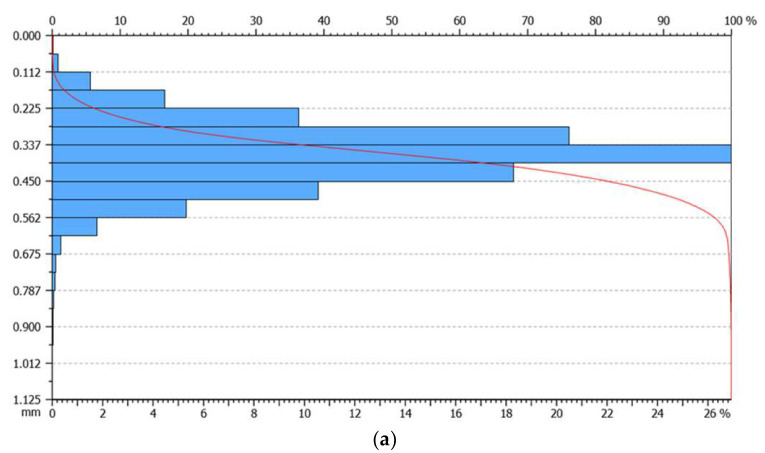

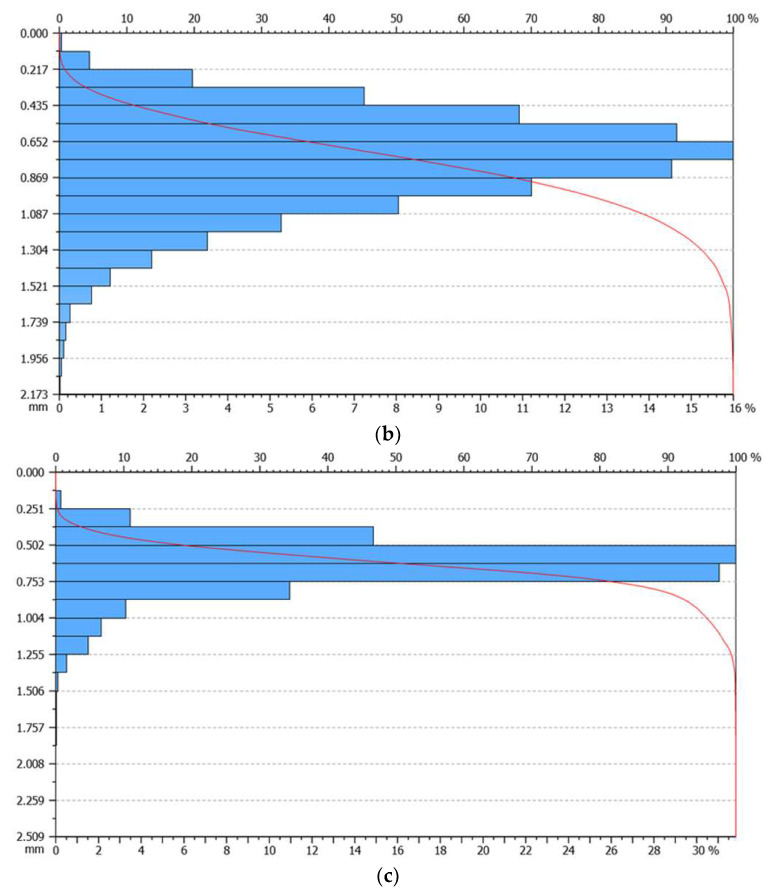
Abbott-Firestone curve for concrete surfaces: unprepared (**a**), sanded (**b**) and grinded (**c**).

**Figure 7:**
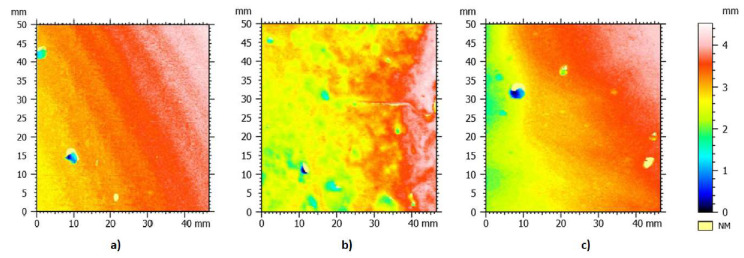
Surface image of samples after scanning with a laser scanner: unprepared (**a**), sanded (**b**) and grinded (**c**).

The Abbott-Firestone curve for the unprepared, sanded and grinded concrete surfaces is presented in Figure 6. When analyzing the obtained values, it can be stated that the material proportions corresponding to the upper (R_mr1_) and lower (R_mr2_) core boundaries are similar for the unprepared and grinded surfaces. In the case of the sanded surface, it should be noted that the material proportion corresponding to the upper limit of the core is more than half, and the lower limit of the core has the lowest value with regards to the other two surfaces. The roughness core depth R_k_ is the most important parameter that says a lot about the nature of the surface. When analyzing the samples macroscopically, it could be clearly stated that the unprepared surface has the smallest roughness, followed by the grinded surface, with the sanded surface having the largest roughness. Scanning studies confirmed this fact by providing Figure 7a–c.

The depth of the middle part of the profile was the smallest for the unprepared surface, and was, on average, 0.21 mm, whereas, for the sanded and grinded surface, it was 0.6935 mm and 0.3556 mm, respectively. The average depth of deep cavities under the roughness profile core was the greatest for the sanded surface (0.3884 mm), decreasing for the grinded surface (0.3427 mm) and reaching the lowest value for the unprepared surface (0.1142 mm). The average height of high mid elevations above the core of the roughness profile was 0.09805 mm, 0.166 mm and 0.1228 mm for the unprepared, sanded and grinded surfaces, respectively. In summary, it can be stated that the highest roughness was found in the sanded surface, and the smallest in the case of the unprepared surface. The grinded surface in this respect is in between the other two. The roughness profile parameters are presented in Table 1.

## 5. Results

**Figure 8:**
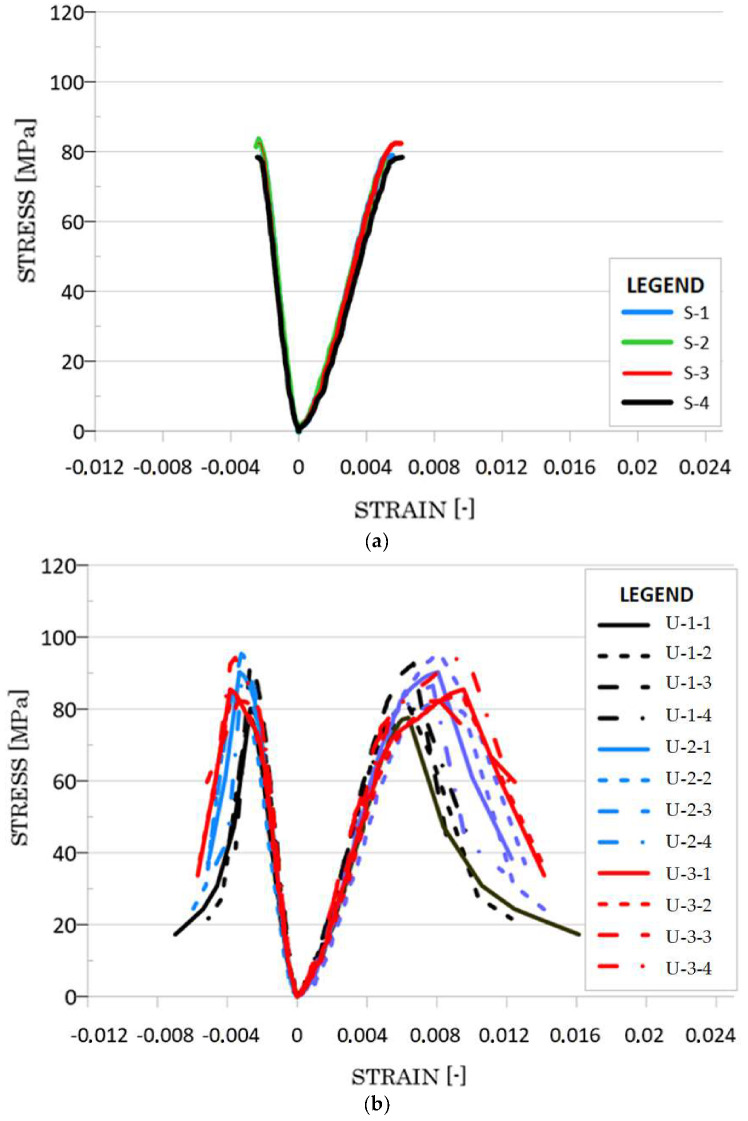

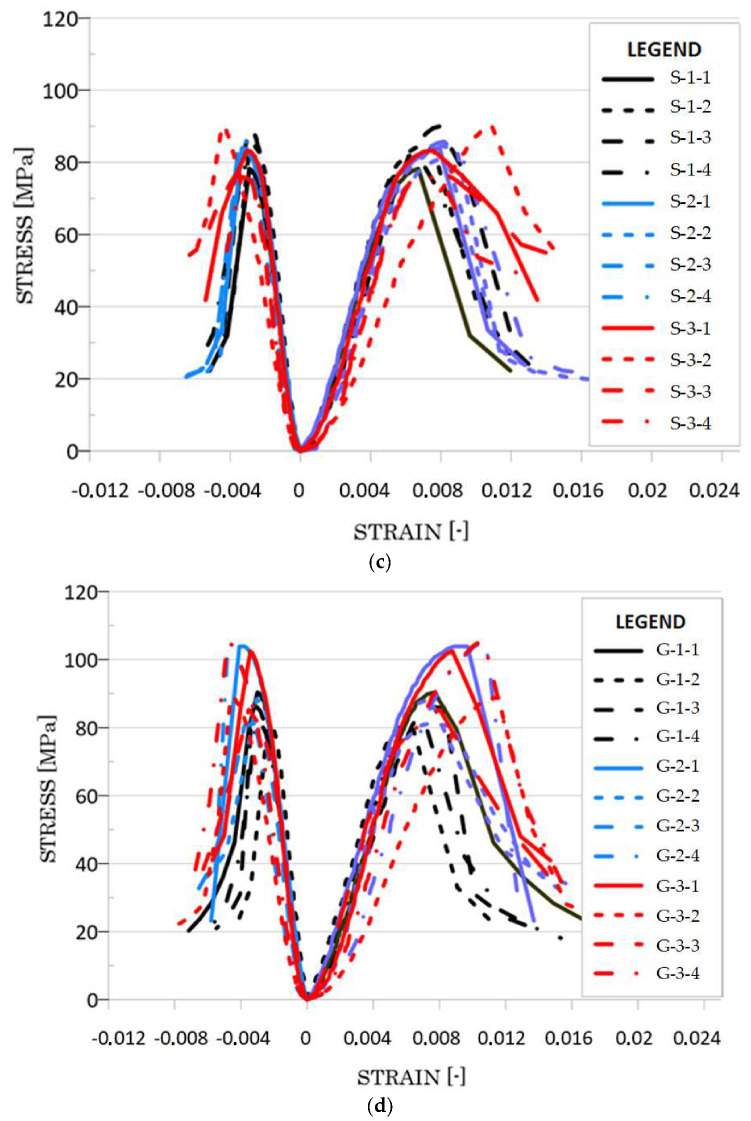
Stress-strain characteristics for: HPSCFRC (**a**); HPSCFRC reinforced carbon fibre reinforced polymer (CFRP) with an unprepared concrete surface (**b**); HPSCFRC reinforced CFRP with a sanded concrete surface (**c**); HPSCFRC reinforced CFRP with grinded concrete surface (**d**).

Detailed results for the samples reinforced with cement matrix are presented in Table 2. In turn, Table 3 presents the average values of the results that were obtained for the HPSCRFC and the group of reinforced samples. The stress—strain characteristics for all samples are presented in Figure 8. For the concrete reinforced with one layer of CFRP, a slight increase in strength was observed, which depended on the type of concrete surface. The average compressive strength was equal to 82.94 MPa, 83.87 MPa and 84.91 MPa for the concretes with an unprepared, sanded and grinded surface, respectively. The average axial deformability of the samples at the moment of obtaining the maximum load capacity was in this case equal to 0.0066, 0.0070 and 0.0072 for the concretes with an unprepared, sanded and grinded surface, respectively. The average lateral deformability of the samples at the same time of failure was 0.0027, 0.0028 and 0.0029, respectively. When analyzing these data, it can be concluded that there is no significant difference between the obtained values of strength and deformability of concrete, with regards to the type of surface preparation. These values differ slightly, and therefore, it can be assumed that the differences are within a statistical error of 5%.

In the case of samples reinforced with two layers of CFRP, a slight increase in compressive strength was observed, and it amounted to 88.69 MPa, 83.67 MPa and 94.79 MPa for the unprepared, sanded and grinded samples, respectively. This means that there is an increase in strength, when compared to non-reinforced concrete, by 10%, 4% and 18%, respectively. The standard deviation of the compressive strength was the lowest for the samples with a sanded surface and amounted to 1.95 MPa. It was higher for the grinded surface (11.89 MPa)-and the unprepared surface (5.44 MPa). The axial deformability of the samples at the moment of the failure of the specimens was equal to 0.0081, 0.0080 and 0.0088 for the concrete with the unprepared, sanded and grinded surface, respectively. The average lateral deformability of the samples was 0.0033, 0.0033 and 0.0038, respectively. For the samples with the grinded concrete surface, the obtained average axial deformation was higher by 9% and 10% when compared to the samples with the unprepared and sanded surfaces. The average transverse deformation was also higher—a 15% increase was recorded for the concrete with a grinded surface, when compared to the samples with the unprepared and sanded surfaces.

For the samples reinforced with three layers of CFRP, a variable value of compressive strength was also found for the samples with the unprepared, sanded and grinded surfaces, and it amounted to 86.34 MPa, 81.82 MPa and 95.57 MPa, respectively. This means that there is an increase in strength when compared to non-reinforced concrete by 7%, 2% and 19%, respectively. The standard deviation of the compressive strength was greatest for the samples with the grinded surface. There was also no change in the average value of the compressive strength of the samples with the sanded surface. The average axial deformability of the samples at the time of failure was equal to 0.0091, 0.0084 and 0.0098 for the concrete with the unprepared, sanded and grinded surfaces, respectively. The lateral deformability of the samples was equal to 0.0036, 0.0035 and 0.0041, respectively. For the grinded surface, the average axial deformability was by 8% and 17% higher than for the samples with the unprepared and sanded surfaces. The average transverse strains were also higher—a 14% and 17% increase for the concrete with the grinded surface was noted, when compared to the samples with the unprepared and sanded surfaces.

### 5.1. The Course of Failure

**Figure 9:**
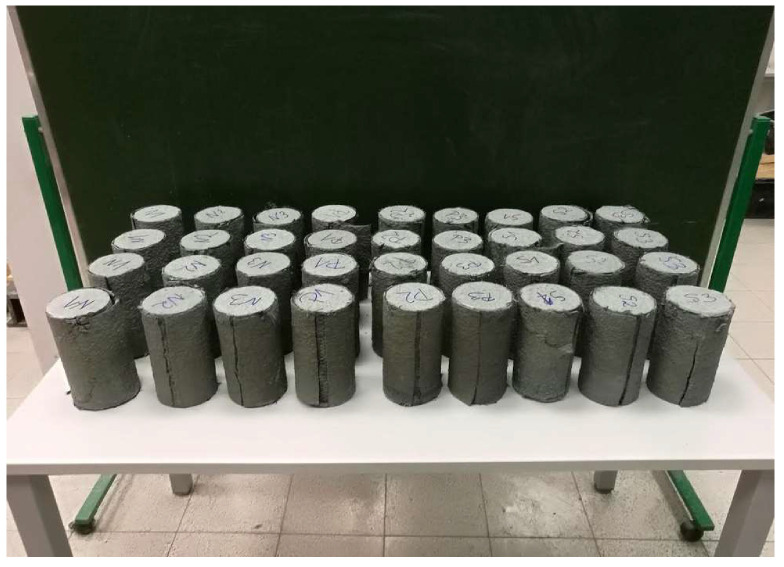
Concrete samples reinforced with carbon fibers using a cement matrix—state after the failure tests.

A macroscopic analysis of the failure of the samples reinforced with one layer of carbon fibers is presented below. In the case of CFRP layers in the amount of more than one, the damage had a similar course, and the adhesion between the first layer of reinforcement and the concrete surface is crucial for the failure process. In all the cases, the overlap was cut. All the samples after the tests are presented in Figure 9.

### 5.2. Unprepared Surface

**Figure 10:**
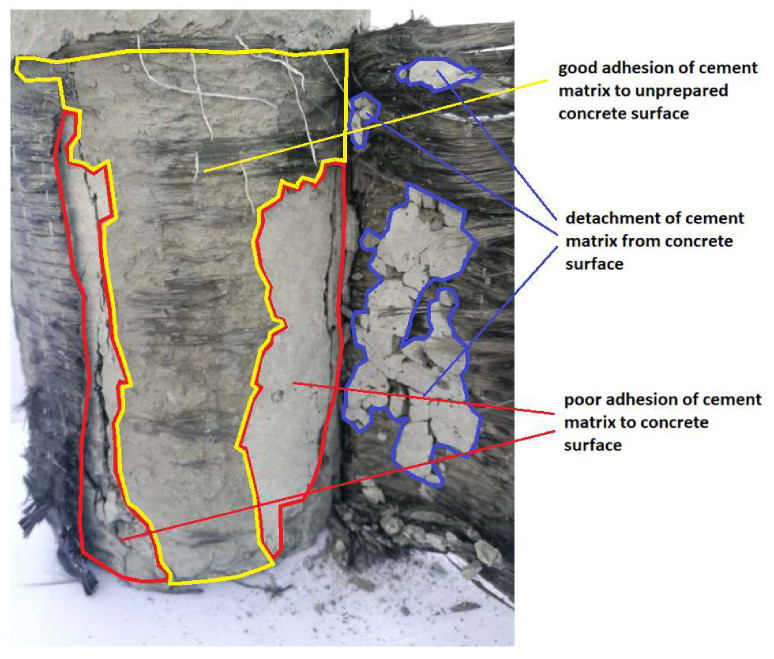
Analysis of the failure of the connection between the carbon fiber reinforced with the cement matrix and the unprepared concrete surface.

In the case of the samples with the unprepared surface, it should be noted that the adhesion of carbon fibers to a high-performance cement matrix is similar to the adhesion of this matrix to the concrete surface. Figure 10 shows the cement matrix, which is detached from the concrete surface (red area), adhering to the carbon fibers in a certain area. The yellow area indicates that the adhesion between the cement matrix and the concrete surface was relatively good. On the surface of the matrix, groups of carbon fibers that are glued to it could be observed locally. This may indicate a local and better infiltration of the carbon fibers with the cement matrix.

### 5.3. Sanded Surface

**Figure 11:**
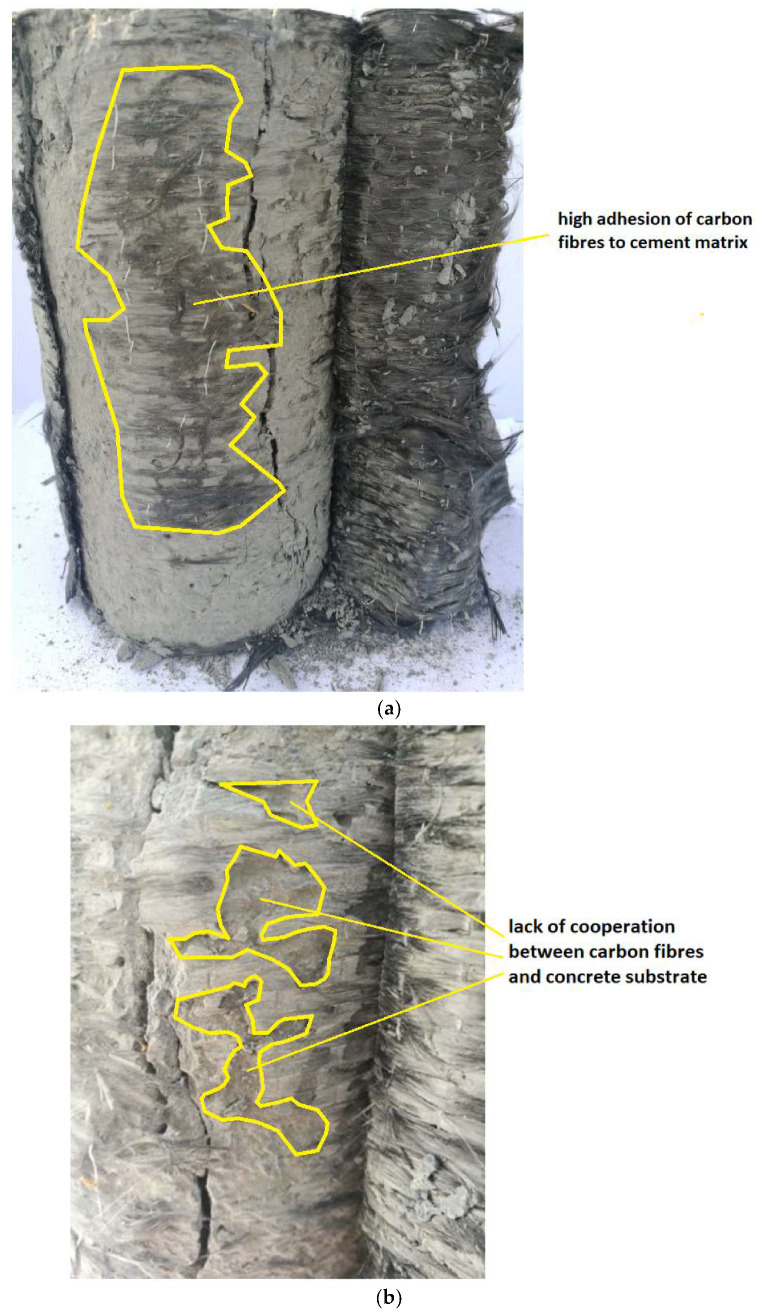
Analysis of the failure of the connection between the carbon fiber reinforced with the cement matrix and the sanded concrete surface: view of the entire sample (**a**) and enlargement of the extrusion area of the cement matrix (**b**).

The better adhesion of the cement matrix to the concrete surface can be observed in the case of the sanded surface. Only a negligible part of the cement matrix remained glued to the carbon fibers—some of them remained on the high-performance matrix during the failure (Figure 11). Locally, places of low adhesion of the matrix to the concrete surface were observed—this could be due to the local pressure of the laminate surface in the place of a larger depression in the surface, which could cause displacement of the fresh mixture, due to its squeezing.

### 5.4. Grinded Surface

**Figure 12:**
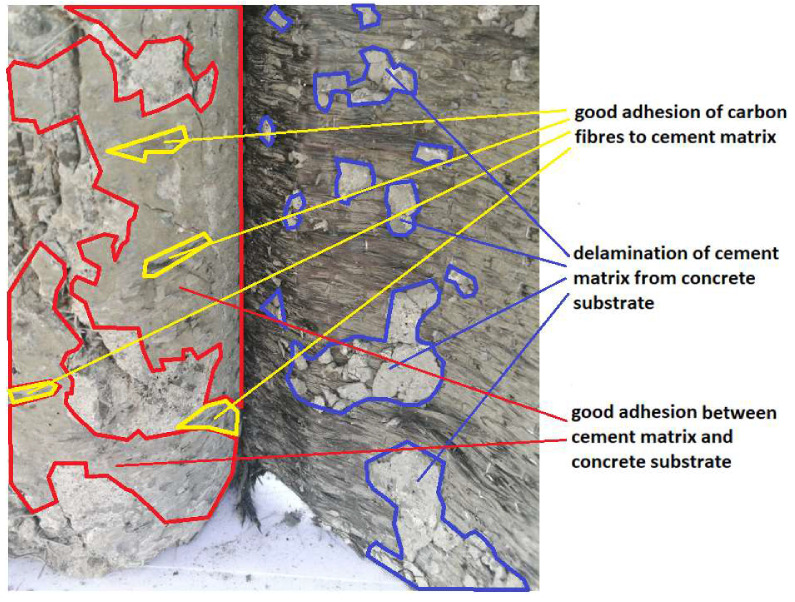
Analysis of the failure of the connection between the carbon fibers reinforced with the cement matrix and the grinded concrete surface.

In the case of the grinded surface, similar phenomena were observed as for the unprepared surface, with the proviso that in this case these phenomena were more dispersed and did not occur on large surfaces. Figure 12 shows the numerous and small areas where the adhesion stresses between the carbon fibers and the cement matrix are greater than between the cement matrix and the concrete surface, and also these areas where the adhesion between the cement matrix and the concrete surface is greater than between the carbon fibers and the cement matrix. A better infiltration of carbon fibers with the cement matrix than in other cases is characteristic for this surface. This may affect the fact that the greatest strengthening efficiency was demonstrated for just the grinded surface.

To sum up, the best cooperation of external reinforcement with a concrete core was demonstrated for the grinded concrete surface. The surface of high-performance concrete is a specific surface, which is due, among other reasons, to its high tightness and low open porosity. Hence, low cement adhesion to the unprepared surface may occur. In the process of strengthening the samples using the considered method, their surface, after the application of the reinforcement, was subjected to some pressure in order to achieve a better infiltration of the fibers. This pressure was made manually by the same person for all the samples. In the case of the sanded surface, it was possible to locally squeeze the fresh cement matrix from the deeper troughs of this surface, which after unloading resulted in the creation of an empty space between the carbon fiber mats and the deeper part of the surface, possible affecting the results. The grinded surface, which has a higher roughness than the unprepared surface, and a smaller roughness than the sanded surface, could have been subjected to the best combination of all the surface types considered, which was confirmed by the results of the compressive strength of the samples.

## 6. Conclusions

The best cooperation of external CF reinforcement with a concrete core was obtained for the grinded concrete surface. The surface of HPSCFRC is a specific surface, due to, among other reasons, its high tightness and low open porosity, when compared to normal performance concrete. Therefore, there could be a low cement mortar adhesion to the unprepared surface. In general, the strength of concrete increases as the number of reinforcing layers increases. However, this regularity was only obtained for the grinded concrete surface. Surface roughness and its morphology significantly affect the obtained results. The lack of preparation of the concrete surface before lamination or too big differences in the height of the surface profile (as in the case of the sanded surface) leads to low cooperation between carbon fibers and the HPSCFRC surface. The most optimal preparation process of concrete substrate is grinding. Given the high cost of CF reinforcement, it is not recommended to use cement matrix, due to low strengthening efficiency, when reinforcing high-strength concrete.

## Figures and Tables

**Table 1:** Roughness profile parameters.

Parameter	Surface U	Surface S	Surface G
R_k_ [mm]	0.21	0.6935	0.3556
R_pk_ [mm]	0.09805	0.166	0.1282
R_vk_ [mm]	0.1142	0.3884	0.3427
R_mr1_ [%]	11.48	8.079	9.953
R_mr2_ [%]	86.17	86.71	87.74

**Table 2:** Detailed results for the samples reinforced with cement matrix.

Specimen	Maximum Compressive Stress [MPa]	Axial Strain During Fracture [–]	Transverse Strain During Fracture [–]
S-1	82.35	0.00604	0.00227
S-2	81.43	0.00630	0.00236
S-3	78.55	0.00604	0.00220
S-4	79.05	0.00633	0.00244
U-1-1	77.63	0.00638	0.00276
U-1-2	81.32	0.00634	0.00263
U-1-3	92.64	0.00665	0.00270
U-1-4	80.16	0.00716	0.00282
U-2-1	90.25	0.00806	0.00331
U-2-2	82.63	0.00829	0.00350
U-2-3	95.38	0.00818	0.00321
U-2-4	86.48	0.00776	0.00321
U-3-1	85.42	0.00954	0.00384
U-3-2	83.46	0.00949	0.00380
U-3-3	82.25	0.00816	0.00338
U-3-4	94.24	0.00919	0.00356
S-1-1	78.20	0.00672	0.00290
S-1-2	84.63	0.00681	0.00261
S-1-3	90.01	0.00790	0.00290
S-1-4	82.65	0.00661	0.00294
S-2-1	83.99	0.00792	0.00332
S-2-2	84.58	0.00811	0.00324
S-2-3	80.85	0.00837	0.00360
S-2-4	85.81	0.00816	0.00310
S-3-1	83.25	0.00758	0.00303
S-3-2	89.99	0.0109	0.00447
S-3-3	78.58	0.00755	0.00332
S-3-4	75.97	0.00798	0.00337
G-1-1	90.37	0.00770	0.00300
G-1-2	80.80	0.00574	0.00215
G-1-3	85.80	0.00829	0.00332
G-1-4	82.66	0.00710	0.00303
G-2-1	103.89	0.00969	0.00410
G-2-2	88.38	0.00745	0.00306
G-2-3	81.26	0.00738	0.00319
G-2-4	105.61	0.01079	0.00478
G-3-1	102.47	0.00879	0.00343
G-3-2	88.88	0.01155	0.00462
G-3-3	85.32	0.00803	0.00353
G-3-4	105.60	0.01067	0.00480

**Table 3:** Average values of the obtained results for the high-performance, self-compacting, fiber-reinforced concrete (HPSCFRC) and samples reinforced with cement matrix.

Matrix	Type of Concrete Surface	Number of CFRP Layers	Average Compressive Strength [MPa]	Standard Deviation of Compressive Strength [MPa]	Average Axial Strain during Fracture [–]	Average Transverse Strain during Fracture [–]
Unreinforced specimens	U	0	80.35	1.83	0.0062	0.0023
Cement matrix	U	1	82.94	6.65	0.0066	0.0027
		2	88.69	5.44	0.0081	0.0033
		3	86.34	5.42	0.0091	0.0036
	S	1	83.87	4.90	0.0070	0.0028
		2	83.67	1.95	0.0080	0.0033
		3	81.82	6.25	0.0084	0.0035
	G	1	84.91	4.19	0.0072	0.0029
		2	94.79	11.89	0.0088	0.0038
		3	95.57	9.97	0.0098	0.0041
